# HBx Downregulates TFEB via the CUL4A/CUL4B–DDB1 Axis to Disrupt Lysosomal Function in Hepatocellular Carcinoma Cells

**DOI:** 10.3390/cells15141259

**Published:** 2026-07-13

**Authors:** Chunyan Zhang, Yuanping Han, Huan Yang

**Affiliations:** 1 School of Basic Medicine, Shaanxi University of Chinese Medicine, Xianyang 712046, China; 2 The Center for Growth, Metabolism and Aging, College of Life Sciences, Sichuan University, Chengdu 610065, China; 3 School of Pharmacy, Zunyi Medical University, Zunyi 563003, China

**Keywords:** HBx, TFEB, lysosome, CUL4A/CUL4B, hepatocellular carcinoma

## Abstract

Hepatitis B virus (HBV) infection remains a major global health burden, with chronic infection leading to severe liver diseases including cirrhosis and hepatocellular carcinoma (HCC). HBV-encoded X protein (HBx) plays a critical role in viral replication and pathogenesis by modulating host cellular processes, including autophagy and lysosomal function. However, the molecular mechanisms by which HBx disrupts lysosomal biogenesis and autophagic degradation remain elusive. In this study, we show that HBx downregulates the transcription factor EB (TFEB), a master regulator of lysosomal biogenesis, which leading to impaired lysosomal acidification and autophagosome–lysosome fusion. Mechanistically, HBx-mediated TFEB downregulation involves the CUL4A (Cullin 4A)/CUL4B (Cullin 4B)-DDB1 (DNA damage-binding protein 1) E3 ubiquitin ligase complex and is dependent on the DDB1-interacting motif in HBx. HBx mutants defective in DDB1 binding (HBx^R96E^ and HBx^ΔDBD^) fail to downregulate TFEB or impair lysosomal function. Collectively, our findings identify a pathway by which HBx disrupts lysosomal function via CUL4A/CUL4B–DDB1-dependent TFEB downregulation, providing insights into HBV-associated liver pathogenesis and highlighting potential targets for therapeutic intervention.

## 1. Introduction

HBV infection remains a significant public health challenge. Worldwide, approximately 254 million people are chronically infected with HBV, with 1.23 million new cases each year [1]. HBV infection is associated with a spectrum of acute and chronic liver diseases—ranging from liver failure and cirrhosis to HCC—and accounts for approximately 1.1 million deaths annually. The HBV genome encodes four proteins: the polymerase (P), surface proteins (pre-S/S), core protein (C), and the X protein (HBx) [2,3]. HBx participates in diverse biological processes, including viral replication, gene transcription, signal transduction and protein degradation [4,5,6]. Studies have shown that HBx activates autophagy [7], a cellular degradation pathway that typically facilitates pathogen clearance. However, it simultaneously impairs late-stage autophagic degradation [8] as evidenced by the accumulation of the autophagy substrate p62 and the disruption of lysosomal acidification. Nevertheless, the precise molecular mechanism underlying HBx-mediated lysosomal dysfunction and autophagic blockade remains elusive.

TFEB, as a master regulator of lysosomal biogenesis, plays a pivotal role in metabolic, inflammatory, and neoplastic diseases [9,10]. In high-fat diet-fed mouse models, TFEB expression levels are inversely correlated with the severity of hepatic steatosis, indicating that TFEB-regulated lysosomal function is essential for maintaining hepatic energy homeostasis [11]. Furthermore, alcohol-fed mice with combined deletion of TFEB and TFE3 (Transcription Factor E3) exhibit markedly exacerbated hepatic steatosis and steatohepatitis [12]. Collectively, these findings underscore the critical involvement of TFEB-driven lysosomal biogenesis in the pathogenesis of liver diseases. Importantly, TFEB protein levels are dynamically regulated by the ubiquitin–proteasome system, and dysregulation of this degradation axis has been implicated in multiple pathological processes. Notably, several E3 ubiquitin ligases and deubiquitinases directly regulate TFEB protein turnover: STIP1 (stress-induced phosphoprotein 1) homology and U-box-containing protein 1 (STUB1) target phosphorylated cytoplasmic TFEB for proteasomal degradation [13]. The IKK–β-TrCP2 axis promotes TFEB degradation through phosphorylation-dependent ubiquitination at a cluster of serine residues (423SPFPSLS429); this motif is recognized by β-TrCP2 [14]. Conversely, USP7 stabilizes TFEB through deubiquitination [15]. These findings establish that TFEB levels are dynamically controlled by ubiquitination events. However, whether viral proteins exploit this regulatory network by recruiting specific E3 ligase complexes to degrade TFEB remains unexplored.

HBx hijacks the host ubiquitin–proteasome system by interacting with the DDB1–CUL4A/CUL4B E3 ubiquitin ligase complex. Structural studies have revealed that HBx binds DDB1 via a conserved H-box motif, inserting an α-helical structure into the BPA–BPC double-propeller domain of DDB1 to function as a viral DCAF (DDB1-CUL4-associated factor) [16]. This interaction is essential for HBV replication, as HBx redirects the Cullin-RING ligase 4 (CRL4) complex to promote ubiquitination and degradation of host restriction factors such as SMC5/6 (Structural Maintenance of Chromosomes 5 and 6) [17]. Notably, the DDB1-binding-deficient HBx mutant (R96E) fails to mediate substrate degradation, confirming the functional dependence on this interaction [16]. Given the established role of HBx as a viral DCAF and its capacity to redirect CRL4-mediated ubiquitination, we hypothesized that HBx may exploit this activity to induce TFEB downregulation.

To test this hypothesis, we investigated the functional consequences of HBx-TFEB interplay in hepatocellular carcinoma cell models. We previously reported that HBx downregulates TFEB [18]. However, the molecular mechanism by which HBx regulates TFEB expression remains poorly understood. The present study aims to elucidate how HBx regulates TFEB expression and thereby modulates autophagic–lysosomal function. Our findings show that HBx downregulates TFEB through the CUL4A/CUL4B–DDB1 axis, which is associated with disrupted lysosomal biogenesis and impaired autophagosome–lysosome fusion. These findings provide novel mechanistic insights into the dysregulation of autophagolysosomal function during the progression of HBV-associated liver diseases.

## 2. Materials and Methods

### 2.1. Reagents and Plasmids

DMEM high-glucose medium was purchased from Hyclone (Logan, UT, USA). Lipofectamine 3000 (Lipo3000) was obtained from Invitrogen (Carlsbad, CA, USA). Puromycin, polybrene, and cycloheximide (CHX) were purchased from Sigma-Aldrich (St. Louis, MO, USA). MG132 and MLN4924 were obtained from Selleck Chemicals (Houston, TX, USA).

The plasmids used in this study were as follows: the lentiviral vector pLVX-puro (Takara Bio USA, Inc., Mountain View, CA, USA), the shRNA vector pLKO.1 (Addgene, Watertown, MA, USA), and the packaging plasmids pMD2.G (Addgene #12259) and psPAX2 (Addgene #12260) (Addgene, Watertown, MA, USA) were preserved in our laboratory. The pCMV-HBx plasmid (Addgene #65463, Watertown, MA, USA) was obtained from Addgene. To generate the pLVX-Flag-HBx construct, the full-length HBx coding sequence was amplified from pCMV-HBx by PCR and inserted into the pLVX-puro vector containing a 3 × Flag tag using standard double-digestion and ligation methods. The pLVX-Flag-HBx^R96E^ and pLVX-Flag-HBx^ΔDBD^ mutant constructs were generated by site-directed mutagenesis using the TOYOBO Site-Directed Mutagenesis Kit (TOYOBO Co., Ltd., Osaka, Japan) following the manufacturer’s instructions, with the pLVX-Flag-HBx plasmid as the template.

For CUL4A and CUL4B knockdown, oligonucleotides encoding the shRNA target sequences were synthesized, annealed to form double-stranded oligos, and cloned into the pLKO.1 vector between the AgeI and EcoRI restriction sites, according to the standard pLKO.1 cloning protocol. The shRNA target sequences were as follows: shCUL4A-1: GCAGAACTGATCGCAAAGCAT; shCUL4A-2: GTGTGGAGAAACAGCTATTAG; shCUL4B-1: GCCATGAAAGAAGCATTTGAA; shCUL4B-2: GCAGAATTTAAAGAGGGTAAA. All constructs were verified by Sanger sequencing (Tsingke Biotechnology Co., Ltd., Beijing, China).

### 2.2. Cell Culture

The human hepatocellular carcinoma cell lines HepG2 and SK-Hep-1, as well as the HEK-293FT cell line, were obtained from the American Type Culture Collection (ATCC, Manassas, VA, USA) [ATCC numbers: HB-8065, HTB-52, and CRL-3216, respectively]. All cell lines were authenticated by the supplier using short tandem repeat (STR) profiling prior to shipment. In our laboratory, cells were used within 10 passages after thawing and routinely confirmed negative for mycoplasma contamination via PCR-based detection using the ATCC^®^ Universal Mycoplasma Detection Kit (ATCC, Manassas, VA, USA). All cells were grown at 37 °C in a humidified 5% CO_2_ atmosphere, using DMEM medium (Hyclone, Logan, UT, USA) supplemented with 10% fetal bovine serum (FBS; Gibco, Grand Island, NY, USA) and 1% penicillin–streptomycin (Gibco, Grand Island, NY, USA).

### 2.3. RNA Extraction and RT-qPCR

Total RNA was isolated from cultured cells using TRIzol reagent (Invitrogen, Carlsbad, CA, USA). Complementary DNA (cDNA) was synthesized with the Hifair II 1st Strand cDNA Synthesis Kit (Yeasen Biotechnology Co., Ltd., Shanghai, China; 11119ES60). RT-qPCR was performed on a Bio-Rad CFX96 Real-Time PCR System (Bio-Rad Laboratories, Hercules, CA, USA) using 2 × Yeasen qPCR Mix (Yeasen Biotechnology Co., Ltd., Shanghai, China). The thermal cycling protocol consisted of an initial denaturation at 95 °C for 2 min, followed by 40 cycles of 95 °C for 10 s, annealing at the optimal temperature for each primer pair (ranging from 58 °C to 62 °C) for 15 s, and extension at 72 °C for 30 s. A melting curve analysis was performed to confirm the specificity of amplification. Reactions were run in triplicate, and relative mRNA expression was calculated by the 2^−ΔΔCt^ method, normalized to GAPDH. Primer sequences were as follows: GAPDH-F: AAAATGGCAGTGCGTTTAG, GAPDH-R: TTTGAAGGCAGTCTGTCGTA; TFEB-F: CCAGAAGCGAGAGCTCACAGAT, TFEB-R: TGTGATTGTCTTTCTTCTGCCG; CUL4A-F: GGACCTCGCACAGATGTACC, CUL4A-R: CGATCGCTGTTCCAAAAGTC; CUL4B-F: GTAGAGTTCGAGGTGGAGTTCAG; CUL4B-R: TTACAATAGTGCTGCCAAATGC.

### 2.4. Construction of Stable Cell Lines

Approximately 24 h before viral packaging, HEK-293FT cells at the exponential growth phase were plated into 6-well dishes at a seeding density of 8 × 10^5^ cells per well. Target vectors (pLVX-puro-based constructs or pLKO.1-shRNA constructs) were co-transfected with packaging vectors (pMD2G and pSPAX2) into HEK-293FT cells using Lipofectamine™ 3000 (Invitrogen, Carlsbad, CA, USA) according to the manufacturer’s protocol. Viral supernatants were harvested 48 h post-transfection and filtered through a 0.45 μm syringe filter (Millipore, Burlington, MA, USA).

For viral infection, target cells were mixed with the filtered virus at a 1:1 (*v*/*v*) ratio, and Polybrene (Sigma-Aldrich, St. Louis, MO, USA) was added to a final concentration of 1 μg/mL. At 24 h post-infection, the medium was replaced with fresh complete medium. After an additional 48 h of culture, cells were trypsinized, centrifuged, and reseeded in medium containing puromycin (Sigma-Aldrich, St. Louis, MO, USA) at a final concentration of 2 μg/mL. Stable cell clones were validated by Western blotting or RT-qPCR 2–3 days post-selection, and all experiments using stable cell lines were performed within 10 passages after selection.

### 2.5. Western Blot Analysis

Cells were harvested and lysed on ice using RIPA buffer (50 mM Tris-HCl pH 7.4, 150 mM NaCl, 1% NP-40, 0.5% sodium deoxycholate, 0.1% SDS) (Beyotime Biotechnology Co., Ltd., Shanghai, China) containing a protease inhibitor cocktail (Roche, Basel, Switzerland). The lysates were vortexed every 5 min for a total of 25 min, followed by centrifugation at 13,000 rpm for 20 min at 4 °C. The supernatant fractions were collected, and protein concentrations were determined using a BCA protein assay kit (Thermo Fisher Scientific, Waltham, MA, USA).

Equal amounts of protein (20–30 μg per lane) were separated by SDS-PAGE using 10% polyacrylamide separating gels, with a 5% stacking gel. Proteins were transferred onto polyvinylidene difluoride (PVDF) membranes (Millipore, Burlington, MA, USA; IPVH00010) using a wet transfer system (Bio-Rad Laboratories, Hercules, CA, USA) at 300 mA constant current for 90 min at 4 °C. After blocking with 5% non-fat milk in Tris-buffered saline with Tween 20 (TBST, prepared in-house) for 1 h at room temperature, membranes were incubated with primary antibodies overnight at 4 °C. The following primary antibodies were used: anti-Flag (mouse monoclonal, 1:2000, F1804; Sigma-Aldrich, St. Louis, MO, USA) and anti-LAMP2A (rabbit polyclonal, 1:1000, ab18528; Abcam, Cambridge, UK). Anti-TFEB (rabbit monoclonal, 1:1000, 37785s), anti-LC3 (rabbit polyclonal, 1:1000, 3869S), and anti-CUL4A (rabbit polyclonal, 1:1000, 2699) were purchased from Cell Signaling Technology (CST) (Danvers, MA, USA). Anti-p62 (rabbit polyclonal, 1:2000, 380612) and anti-CUL4B (rabbit polyclonal, 1:1000, 20882-1-AP) were purchased from Proteintech (Proteintech Group, Inc., Rosemont, IL, USA), anti-GAPDH (mouse monoclonal, 1:5000, 200306), and anti-β-Actin (mouse monoclonal, 1:5000, 200068) were obtained from Zen Bioscience Co., Ltd. (Chengdu, China). Following three washes with TBST, membranes were incubated with horseradish peroxidase (HRP)-conjugated secondary antibodies (goat anti-mouse or goat anti-rabbit, 1:5000, ZSGB-BIO Co., Ltd., Beijing, China) for 1 h at room temperature. Protein bands were visualized using an enhanced chemiluminescence (ECL) detection system (Thermo Fisher Scientific, Waltham, MA, USA). Densitometric quantification was performed using ImageJ software (version 1.53t, National Institutes of Health, Bethesda, MD, USA). All Western blotting experiments were performed with three independent biological replicates.

### 2.6. LysoTracker Staining

HepG2 or SK-Hep-1 cells were trypsinized, centrifuged at room temperature (1000 rpm for 5 min), and seeded onto glass coverslips in 12-well plates at a density of 1 × 10^4^ cells per well. Following 24 h of attachment, cells were exposed to medium supplemented with 50 nM LysoTracker™ Red DND-99 (Invitrogen, Carlsbad, CA, USA) and Hoechst 33342 (Sigma-Aldrich, St. Louis, MO, USA; diluted 1:1000) for 1 h at 37 °C. After staining, coverslips were gently washed three times with phosphate-buffered saline (PBS; prepared in-house), mounted onto glass slides with anti-fade mounting medium (Beyotime Biotechnology Co., Ltd., Shanghai, China), and observed under a laser scanning confocal microscope (LSM 880, Zeiss, Oberkochen, Germany). Images were captured and analyzed using ZEN 2.3 software (Carl Zeiss AG, Oberkochen, Germany).

### 2.7. LC3-GFP-RFP Dual Fluorescent Probe Assay

Cells stably expressing the LC3-GFP-RFP tandem fluorescent reporter plasmid (pBABE-puro-mRFP-GFP-LC3, Addgene #22418, Watertown, MA, USA), which enables discrimination between autophagosomes (yellow, GFP^+^/RFP^+^) and autolysosomes (red-only, GFP^−^/RFP^+^), were used to evaluate autophagic flux. Cells stably expressing LC3-GFP-RFP (alone or co-expressing HBx) were seeded onto glass coverslips in 24-well plates and cultured for 24 h. After washing with PBS, coverslips were mounted with anti-fade mounting medium (Beyotime Biotechnology Co., Ltd., Shanghai, China) and imaged using a laser scanning confocal microscope (LSM 880, Carl Zeiss AG, Oberkochen, Germany). The numbers of autophagosomes (GFP^+^/RFP^+^ double-positive puncta) and autolysosomes (GFP^−^/RFP^+^ puncta) were counted using ImageJ software (version 1.53t, National Institutes of Health, Bethesda, MD, USA). At least 50 cells were analyzed per group.

### 2.8. Mutant Construction

Site-directed mutagenesis and deletion mutants of HBx were constructed using the TOYOBO Site-Directed Mutagenesis Kit (Cat. No. SMK-101, TOYOBO Co., Ltd., Osaka, Japan) following the manufacturer’s instructions. Primers were designed to introduce specific mutations or deletions (sequences available upon request) and synthesized by Tsingke Biotechnology Co., Ltd. (Beijing, China). The mutagenesis procedure included: (1) inverse PCR amplification of the target plasmid; (2) digestion of the parental plasmid with DpnI restriction enzyme (Thermo Fisher Scientific, Waltham, MA, USA) to eliminate template DNA; (3) self-ligation of the PCR product using T4 DNA ligase (Thermo Fisher Scientific, Waltham, MA, USA); (4) transformation of the ligation product into competent *E. coli* DH5α cells (Tsingke Biotechnology Co., Ltd., Beijing, China); and (5) identification of positive clones by Sanger sequencing (Tsingke Biotechnology Co., Ltd., Beijing, China).

### 2.9. Statistical Analysis

All data are expressed as mean ± standard deviation (SD) and were analyzed with GraphPad Prism 7.0 software (GraphPad Software, Inc., La Jolla, CA, USA). Each experiment was performed in triplicate with 2–3 technical replicates per group. Statistical significance between two groups was determined using unpaired two-tailed Student’s t-test. For comparisons among multiple groups, one-way ANOVA with Dunnett’s post-hoc test was used. Differences were considered statistically significant at * *p* < 0.05, ** *p* < 0.01, and *** *p* < 0.001; ns indicated no significant difference (*p* ≥ 0.05).

## 3. Results

### 3.1. HBx Downregulates TFEB and Impairs Lysosomal Function

**Figure 1:**
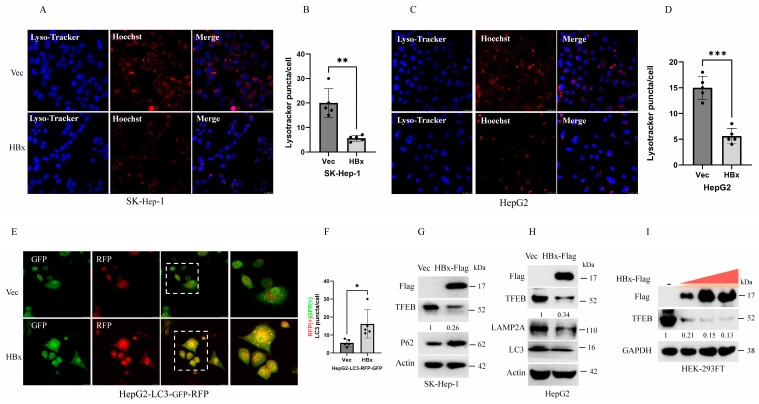
HBx Downregulates TFEB and Impairs Lysosomal Function. Representative confocal images of LysoTracker (red, acidic lysosomes) and Hoechst (blue, nuclei) staining in SK-Hep-1 (**A**) and HepG2 (**C**) cells stably expressing vector control (Vec) or HBx. (**B**,**D**) Quantification of LysoTracker puncta per cell from (**A**) and (**C**), respectively. (**E**) Representative confocal images of HepG2 cells stably expressing the LC3-GFP-RFP tandem reporter, showing GFP-LC3 (green, autophagosomes), RFP (red, autolysosomes), and merged images (yellow indicates colocalization), with or without HBx co-expression. Dashed white boxes indicate the regions magnified in the rightmost panels. (**F**) Quantification of the ratio of yellow puncta to total LC3 puncta per cell from (**E**). (**G**) Western blot analysis of TFEB and p62 protein levels in SK-Hep-1 cells stably expressing Vec or HBx. (**H**) Western blot analysis of TFEB, LAMP2A, and LC3 in HepG2 cells stably expressing Vec or HBx. (**I**) HEK-293FT cells were transfected with increasing amounts of HBx-Flag plasmid (0, 0.5, 1.0, and 2.0 μg; indicated by the red gradient triangle). Western blot analysis for TFEB, Flag, and GAPDH. Data are presented as mean ± SD from three independent experiments (n ≥ 50 cells per group). * *p* < 0.05, ** *p* < 0.01, and *** *p* < 0.001 (unpaired two-tailed Student’s *t*-test). Scale bar = 25 µm.

To explore how HBx disrupts lysosomal function, LysoTracker staining was carried out. As shown in Figure 1A–D, overexpression of HBx significantly impaired lysosomal acidification in SK-Hep-1 or HepG2 cells. The autophagolysosomal pathway requires the fusion of autophagosomes with lysosomes to exert its substrate degradation function. The LC3-GFP-RFP dual-fluorescence labeling plasmid is a classic tool for evaluating the autophagolysosomal pathway [19]. In HepG2 cells overexpressing HBx, GFP signal persisted in LC3 puncta, resulting in a significant increase in yellow puncta (GFP-RFP co-localized) compared to the control group (Figure 1E,F), indicating impaired autophagosome-lysosome fusion. TFEB, a master transcription factor of lysosomal biogenesis, translocates to the nucleus and binds to the coordinated lysosomal expression and regulation (CLEAR) network to promote transcription of lysosomal and autophagic genes [9]. In SK-Hep-1 cells overexpressing HBx, the protein levels of TFEB were markedly decreased, whereas p62 was accumulated (Figure 1G). Similarly, in HepG2 cells, HBx overexpression also decreased TFEB and LAMP2A levels (Figure 1H). Furthermore, in HEK-293FT cells, TFEB was downregulated in a dose-dependent manner with increasing expression of HBx (Figure 1I). Collectively, these results demonstrate that HBx disrupts autophagosome-lysosome fusion and reduces lysosomal biogenesis, which is associated with downregulation of TFEB.

### 3.2. HBx Promotes TFEB Degradation via the Proteasomal Pathway

**Figure 2:**
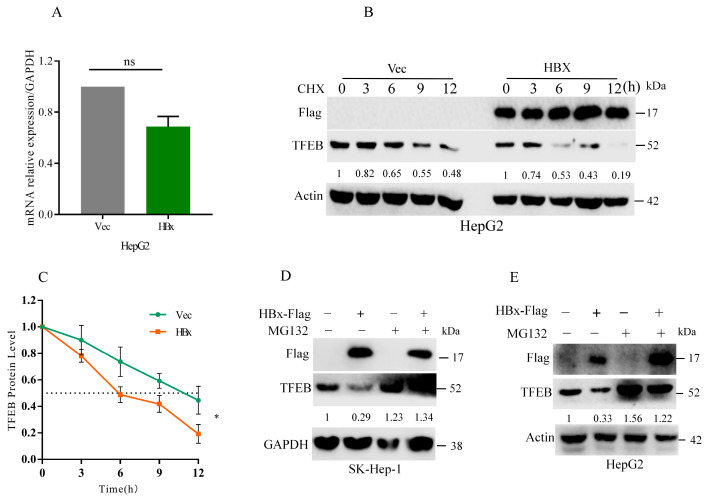
HBx Promotes TFEB Degradation via the Proteasomal Pathway. (**A**) Quantitative RT-PCR analysis of TFEB mRNA expression in HepG2 cells stably expressing Vec or HBx (unpaired two-tailed Student’s *t*-test). (**B**) HepG2 cells stably expressing Vec or HBx were treated with cycloheximide (CHX, 20 μg/mL) for the indicated time points (0, 3, 6, 9, and 12 h). TFEB protein levels were analyzed by Western blotting. (**C**) Quantification of TFEB protein remaining at each time point relative to time 0 (set as 100%) from (**B**). The dashed line indicates the half-life threshold (50% of initial TFEB protein level). One-way ANOVA with Dunnett’s post-hoc test. (**D**,**E**) SK-Hep-1 (**D**) or HepG2 (**E**) cells stably expressing HBx were treated with or without 10 μM MG132 for 6 h, followed by Western blotting for TFEB. Data are mean ± SD from three independent experiments. * *p* < 0.05, ns, not significant; *p* ≥ 0.05.

To investigate whether HBx regulates TFEB at the transcriptional level, we first examined TFEB mRNA expression in HepG2 cells stably expressing HBx. No significant change was observed in TFEB mRNA levels upon HBx overexpression, suggesting the regulation occurs at the protein level (Figure 2A). Next, we assessed TFEB protein stability by cycloheximide (CHX) chase assay. As shown in Figure 2B,C, the half-life of TFEB was markedly shortened in HBx-overexpressing HepG2 cells compared with vector controls, indicating that HBx accelerates TFEB turnover. As the ubiquitin-proteasome system represents a major protein degradation pathway, we next examined whether HBx-mediated TFEB downregulation is proteasome-dependent. Treatment with MG132, a specific proteasome inhibitor, effectively rescued the HBx-induced reduction in TFEB in SK-Hep-1 or HepG2 cells (Figure 2D,E). Collectively, these results demonstrate that HBx promotes TFEB degradation through the proteasomal pathway.

### 3.3. CUL4A/CUL4B Mediate HBx-Induced TFEB Degradation

**Figure 3:**
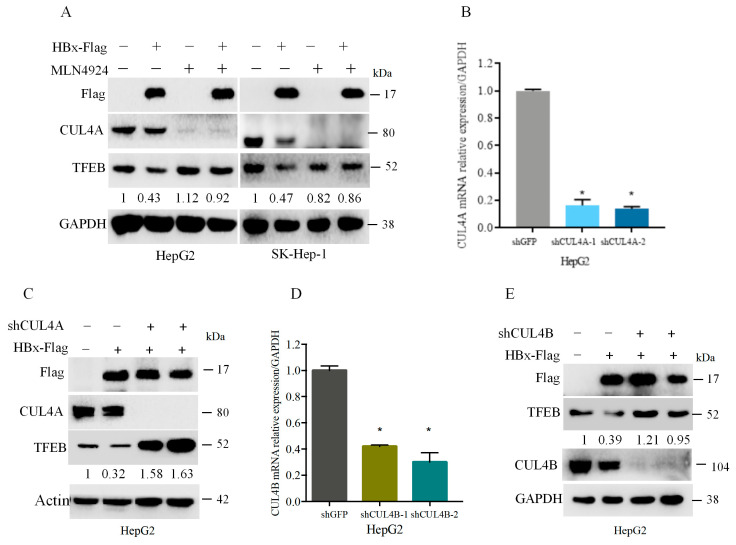
CUL4A/CUL4B Mediate HBx-Induced TFEB Degradation. (**A**) HepG2 or SK-Hep-1 cells stably expressing HBx or Vec were treated with 10 nM MLN4924 or DMSO for 12 h, followed by Western blotting for TFEB and Flag. (**B**) HepG2 cells with shGFP or shCUL4A knockdown were subjected to RT-qPCR for CUL4A (normalized to GAPDH). (**C**) HepG2 cells stably expressing HBx were infected with lentivirus encoding shCUL4A, followed by Western blotting analyses for TFEB, CUL4A, and Flag. (**D**) HepG2 cells with shGFP or shCUL4B knockdown were subjected to RT-qPCR for CUL4B (normalized to GAPDH). (**E**) HepG2 cells stably expressing HBx were infected with lentivirus encoding shCUL4B, followed by Western blotting analyses for TFEB, CUL4B and Flag. Each experiment was repeated three times. Statistical significance was determined by unpaired two-tailed Student’s *t*-test (* *p* < 0.05).

Prior studies have demonstrated that HBx hijacks the DDB1-CUL4A/CUL4B complex to promote target substrate degradation [17,20]. CUL4A and CUL4B, as core subunits of the CRL4 E3 ubiquitin ligase complex, mediate the proteasomal degradation of various substrates. We therefore hypothesized that CUL4A/CUL4B mediate HBx-induced TFEB degradation. MLN4924, an NEDD8-activating enzyme inhibitor, blocks cullin neddylation, thereby inactivating CUL4A/CUL4B E3 ligase activity [21]. As shown in Figure 3A, MLN4924 treatment significantly attenuated HBx-induced TFEB degradation in HepG2 or SK-Hep-1 cells. To further confirm the involvement of CUL4A, we knocked down CUL4A using shRNA. Efficient knockdown was verified by qPCR (Figure 3B). TFEB protein levels were restored following CUL4A silencing (Figure 3C). We next established CUL4B-knockdown cells. qPCR confirmed efficient CUL4B mRNA downregulation (Figure 3D). Consistent with CUL4A knockdown results, TFEB protein levels were significantly rescued upon CUL4B silencing (Figure 3E). Taken together, these findings demonstrate that CUL4A/CUL4B mediate HBx-induced TFEB degradation.

### 3.4. The DDB1-Binding Activity of HBx Is Required for CUL4A/CUL4B-Mediated TFEB Degradation

**Figure 4:**
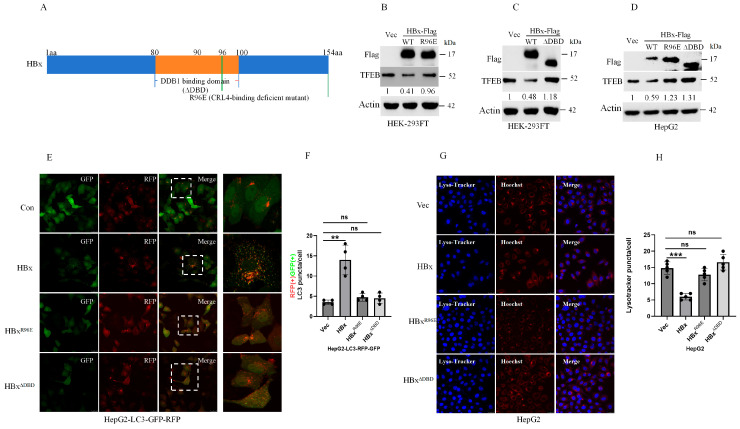
The DDB1-Binding Activity of HBx Is Required for CUL4A/CUL4B-Mediated TFEB Degradation. (**A**) Schematic diagram of the HBx protein structure (1–154 aa). Blue indicates the full-length HBx protein; orange indicates the DDB1-binding domain (ΔDBD); green indicates the R96E mutation site (CRL4-binding deficient mutant). Key amino acid positions are numbered. (**B**,**C**) HEK-293FT cells were transfected with vector, or HBx^WT^-Flag, or HBx^R96E^-Flag, or HBx^∆DBD^-Flag plasmids, followed by Western blotting analyses for TFEB and Actin. (**D**) HepG2 cells stably expressing vector, or HBx^WT^-Flag, HBx^R96E^-Flag, or HBx^∆DBD^-Flag plasmids were subjected to Western blotting for TFEB and Actin. (**E**) HepG2-LC3-GFP-RFP cells expressing the indicated constructs were imaged by confocal microscopy. Dashed white boxes indicate the regions magnified in the rightmost panels. (**F**) Quantification of the ratio of yellow puncta to total LC3 puncta per cell from (**E**). (**G**) HepG2 cells expressing the indicated constructs were stained with LysoTracker (red, acidic lysosomes) and Hoechst (blue, nuclei), then imaged by confocal microscopy. (**H**) Quantification of LysoTracker puncta per cell from (**G**). Data are presented as mean ± SD from three independent experiments (n ≥ 50 cells per group)., ** *p* < 0.01, and *** *p* < 0.001, ns, not significant. One-way ANOVA with Dunnett’s post-hoc test. Scale bar = 25 µm.

Studies have reported that wild-type (WT) HBx binds to the CUL4A/CUL4B/DDB1 complex to form a stable assembly, thereby mediating the degradation of target substrates [22]. In contrast, HBx^R96E^ loses the ability to interact with DDB1, which in turn prevents the assembly of the CUL4A/CUL4B-DDB1 E3 ubiquitin ligase complex and abrogates the complex’s capacity to mediate substrate degradation [23]. Structural analyses further identified that HBx interacts with DDB1 through a region spanning residues 80–100 [16]. A schematic diagram of HBx and its mutants is shown in Figure 4A. To verify whether the DDB1-CUL4A/CUL4B interaction is required for TFEB degradation, we performed transient transfection assays in HEK-293FT cells using plasmids encoding HBx^WT^, HBx^R96E^, or HBx^ΔDBD^. Compared with HBx^WT^, the HBx mutants were unable to downregulate TFEB protein levels, as shown in Figure 4B,C. To further confirm this, we established HepG2 cell lines stably expressing HBx^R96E^ or HBx^∆DBD^. Consistent with the transient transfection results, stable expression of either mutant also failed to reduce TFEB expression (Figure 4D). We next assessed whether the DDB1-binding defect affects downstream lysosomal and autophagic functions. In HepG2-LC3-GFP-RFP cells, HBx^WT^ induced a significant increase in yellow puncta, indicating impaired autophagosome-lysosome fusion. In contrast, both HBx^R96E^ and HBx^ΔDBD^ failed to induce this phenotype, with GFP signal being efficiently quenched (Figure 4E,F). Similarly, LysoTracker staining revealed that HBx^WT^ impaired lysosomal acidification, an effect abolished by the HBx^R96E^ and HBx^ΔDBD^ mutations (Figure 4G,H). Taken together, these findings demonstrate that the DDB1-binding activity of HBx is essential for CUL4A/CUL4B-mediated TFEB degradation.

## 4. Discussion

HBx, a critical regulatory protein encoded by HBV, has been reported to induce autophagy while simultaneously impairing lysosomal acidification, thereby blocking autophagic degradation and promoting viral persistence [8]. However, the underlying molecular mechanisms remain incompletely understood. In the present study, we identify a novel mechanism wherein HBx downregulates TFEB via the CUL4A/CUL4B–DDB1 axis to disrupt lysosomal function.

TFEB is a master transcription factor that coordinates the autophagy–lysosome pathway. Our findings demonstrate that HBx overexpression leads to significant accumulation of the autophagy substrate p62, indicative of impaired autophagic flux. Furthermore, we reveal that HBx attenuates lysosomal degradative function by promoting TFEB degradation. This is particularly significant given that functional lysosomes are required for the degradation of HBV components [24]. By disrupting this process, HBx not only evades clearance but also exacerbates HBV-induced cellular pathogenesis.

The involvement of the CRL4 E3 ligase complex in HBx function is well-documented. HBx mimics a cellular DCAF to hijack the ubiquitin-proteasome system and degrade host restriction factors [25]. CUL4 proteins utilize DDB1 as a substrate receptor to recognize and bind a variety of target proteins [26]. Additionally, HBx has been shown to bind to the CUL4A/CUL4B/DDB1 complex to form a stable assembly, thereby mediating substrate degradation [22]. Our findings demonstrate that HBx mutants defective in DDB1 binding (HBx^R96E^ and HBx^ΔDBD^) are unable to induce TFEB degradation or disrupt lysosomal and autophagic functions, supporting the requirement of the DDB1-CUL4A/CUL4B interaction for HBx-mediated TFEB downregulation. Direct evidence of TFEB ubiquitination by the HBx-recruited CRL4 complex, however, remains to be established, as do physical interactions among HBx, DDB1-CUL4A/CUL4B, and TFEB demonstrable by co-immunoprecipitation or pull-down assays. Future studies employing in vivo ubiquitination systems will be essential to confirm ternary complex formation.

In summary, this study elucidates a mechanism by which HBx, through the DDB1-CUL4A/CUL4B axis, downregulates TFEB, leading to defective lysosomal acidification and impaired autophagic flux. It provides insights into HBV-associated liver pathogenesis and highlights potential targets for therapeutic intervention. Future studies should address whether the HBx-TFEB axis contributes to hepatocarcinogenesis in HBV-infected liver tissues.

## Data Availability

The original contributions presented in this study are included in the article/Supplementary Materials. Further inquiries can be directed to the corresponding author.

## Supplementary Materials

The following supporting information can be downloaded at: https://www.mdpi.com/article/10.3390/cells15141259/s1. Figure S1. Uncropped Western blot images corresponding to Figure 1. (A) Full-length blot for TFEB, Flag, p62 and Actin (from Figure 1G). (B) Full-length blot for TFEB, Flag, LAMP2A, LC3 and Actin (from Figure 1H). (C) Full-length blot for TFEB, Flag, and GAPDH (from Figure 1I). Figure S2. Uncropped Western blot images corresponding to Figure 2. (A) Full-length blot for TFEB, Flag, and Actin (from Figure 2B). (B) Full-length blot for TFEB, Flag, and GAPDH (from Figure 2D). (C) Full-length blot for TFEB, Flag, and Actin (from Figure 2E). Figure S3. Uncropped Western blot images corresponding to Figure 3. (A) Full-length blot for TFEB, CUL4A, Flag, and GAPDH (from Figure 3A-HepG2). (B) Full-length blot for TFEB, CUL4A, Flag, and GAPDH (from Figure 3A-SK-Hep-1). (C) Full-length blot for TFEB, CUL4A, Flag, and Actin (from Figure 3C). (D) Full-length blot for TFEB, CUL4B, Flag, and Actin (from Figure 3E). Figure S4. Uncropped Western blot images corresponding to Figure 4. (A) Full-length blot for TFEB, Flag, and Actin (from Figure 4B,C). (B) Full-length blot for TFEB, Flag, and Actin (from Figure 4D).
